# Are Vulnerable Communities Thoroughly Informed on Mosquito Bio-Ecology and Burden?

**DOI:** 10.3390/ijerph17218196

**Published:** 2020-11-06

**Authors:** Mmabaledi Buxton, Honest Machekano, Nonofo Gotcha, Casper Nyamukondiwa, Ryan J. Wasserman

**Affiliations:** Department of Biological Sciences and Biotechnology, Botswana International University of Science and Technology, P. Bag 16, Palapye, Botswana; machekanoh@biust.ac.bw (H.M.); nonofo.gotcha@studentmail.biust.ac.bw (N.G.); nyamukondiwac@biust.ac.bw (C.N.); ryanwas21@gmail.com (R.J.W.)

**Keywords:** Central district Botswana, emerging-re-emerging diseases, knowledge attitude practice (KAP), mosquito-borne infection, vector-borne diseases

## Abstract

Mosquitoes account for a significant burden of morbidity and mortality globally. Despite evidence of (1) imminent anthropogenic climate and environmental changes, (2) vector-pathogen spatio-temporal dynamics and (3) emerging and re-emerging mosquito borne infections, public knowledge on mosquito bio-ecology remain scant. In particular, knowledge, attitude and practices (KAPs) on mosquitoes are often neglected despite otherwise expensive remedial efforts against consequent infections and other indirect effects associated with disease burden. To gather baseline KAPs that identify gaps for optimising vector-borne disease control, we surveyed communities across endemic and non-endemic malaria sub-districts (Botswana). The study revealed limited knowledge of mosquitoes and their infections uniformly across endemic and non-endemic areas. In addition, a significant proportion of respondents were concerned about mosquito burdens, although their level of personal, indoor and environmental protection practices varied significantly across sub-districts. Given the limited knowledge displayed by the communities, this study facilitates bridging KAP gaps to minimise disease burdens by strengthening public education. Furthermore, it provides a baseline for future studies in mosquito bio-ecology and desirable control practices across differential spheres of the rural–urban lifestyle, with implications for enhanced livelihoods as a consequence of improved public health.

## 1. Introduction

Vector-borne infections contribute significantly to human morbidity and mortality globally [1], accounting annually for about one million deaths and ~17% of the overall infectious disease burden worldwide [2]. Whilst several arthropods are implicated in a wide range of public health epidemiological systems [3,4,5], vector mosquitoes have accounted for the majority of disease burdens [6,7]. Mosquitoes harbor pathogens (e.g., *Plasmodium* parasites, arboviruses, nematodes) that are causative agents for debilitating human, wildlife and livestock diseases [8,9,10]. Although the distribution of vector mosquitoes and associated diseases is generally explored in Africa [11,12], some areas remain underexplored owing to the lag between unreported species and invasion potentials under current global change scenarios [13]. Many of these vectors and pathogens exploit biotic and abiotic systems (e.g., hosts, climate, transportation systems and environmental modifications) across rural–urban landscapes to proliferate [14,15,16]. 

The extent to which these factors contribute to vector-parasite establishment in novel environments have been explored effectively in many parts of the world [17], however little attention has been given to arid and semi-arid environments (e.g., Botswana). This knowledge paucity hinders information dissemination in communities exposed to risks of emerging and re-emerging diseases although control efforts and educational programmes may be restricted to hotspot zones with little or no extension beyond these areas [18]. Amongst the mosquito-borne diseases, malaria is of greatest epidemiological concerns globally [19]. Malaria cases are currently estimated at 228 million worldwide with most occurring in the African region (93%), followed by the South-East Asia region (3.4%) [20]. Although malaria incidences are known to be on the decline [21], sub-Saharan Africa still exhibits the highest deaths rates with pregnant women and children under five as vulnerable groups [22]. 

In the semi-arid Botswana, the only apparent mosquito-borne disease is malaria, endemic to the northern part of the country [23]. Previous knowledge, attitude and practices (KAPs) studies have shown that communities from endemic areas (Okavango) were well informed on the dynamics of malaria transmission [18]. Currently, the country is at an elimination stage with intensified surveillance and vector control intervention strategies [24], however the level of information dissemination, aimed at empowering communities in both endemic and non-endemic areas, is unknown. Non-endemic regions adjacent to endemic regions are vulnerable to spread of diseases associated with climate change and vector invasion mechanisms [25,26]. Whilst research on dynamics of mosquito-borne infections is evident in the country’s malaria endemic areas [27,28,29], Serowe, Palapye and other non-endemic region across the country remain neglected. Nevertheless, assessment of mosquito KAPs and regular monitoring of vector-pathogen burdens capable of extending in range are also critical in these regions. Given the proximity of these non-endemic sub-districts to the endemic zones, against the backdrop of anthropogenic climate change [30,31], shifting vector-pathogens [32] and increased emerging re-emerging disease incidences [33], more local contextual work on mosquitoes and associated infection baseline studies are warranted (although see [34,35]). 

The country’s national vector control programme deployed chemical-mediated approaches for vector management since the 1950s [36]. As this intervention has been going on in endemic zones for decades, no published work reports vector status on mosquitoes although some studies have reported insecticide resistance to commonly used insecticides worldwide [37]. Apart from a few studies based only on the northern part of the country [29,38], the knowledge on diversity and distribution of vector mosquitoes across the country is not well documented [29,34,35]. In light of the national mosquito diversity spectrum, non-endemic areas remain a priority considering the recent changing environments [31] and presence of highly invasive species [13]. 

Mosquitoes selectively breed in diverse aquatic systems that may even consist of compromised water quality (e.g., polluted, highly turbid, bogs, marshes or brackish waters) across different habitats [39,40]. Given this behavioral adaptation, colonisation in Botswana’s rural–urban arid and semi-arid landscapes may be possible. This may be encouraged by natural, modified and artificial wetland structures that hold water, serving as “breeding hotspots”. Most of these have been implicated locally as aquatic ecosystems that potentially promote larval mosquito infestation and adult proliferation. The country’s national vector control strategic plan mainly targets the mosquito adults through indoor residual spraying (IRS) and long-lasting insecticide-treated nets (LLITNs) [41] and largely neglect the control of aquatic life stages (eggs, larvae, pupae). However, recent work has suggested that compromised aquatic habitats, support fewer aquatic predators and favour larval mosquito proliferation [40,42,43]. As such, more work is essential in managing the mosquito aquatic stages from wetland systems through desirable and sustainable eco-friendly approaches (e.g., natural enemies) [44,45]. This may be a pivotal alternative, used in an integrated approach for mosquito population reduction in the country. In addition, the KAPs on mosquito ontogeny and life-history traits, mosquito-borne disease prevention and control dynamics are not yet assessed in many communities across the endemic and non-endemic zones (although see [18]). In this regard, KAPs evaluate public knowledge level and have the potential to strengthen individuals, households and communities against escalating disease burdens at various scales. Furthermore, KAPs are key in empowering end users on ways to deal with issues that directly concern public health through community engagement for the management of disease prevention and spread. Overall, KAPs play a pivotal role in optimising community health programme planning and serve as points in health system reforms [46]. 

This work was aimed at assessing mosquito KAPs on communities of endemic and non-endemic semi-arid Botswana, exploring areas that may help bridging knowledge gaps in vector and associated disease dynamics. The results of this study benchmark community level KAPs serving as baseline for building future research and interventions aimed at reducing disease burden through empowering communities. Here, we hypothesised that (1) communities in malaria endemic areas are better informed on mosquito related issues and that (2) communities across study sites protect themselves against mosquitoes differentially, due to disease burden bias in malaria endemic incriminated sub-districts.

## 2. Materials and Methods

### 2.1. Knowledge Attitude and Practices

**Figure 1:**
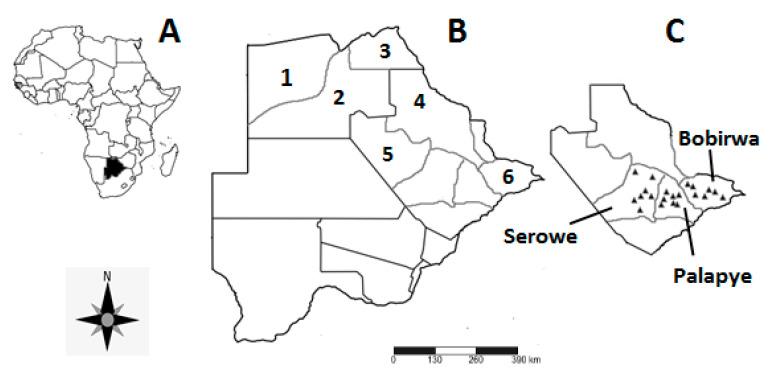
Map showing (**A**) the location of Botswana within Africa, (**B**) the malaria endemic sub-districts and the study site in Botswana, and (**C**) surveyed villages of the Central district; Serowe, Palapye and Bobirwa sub-districts. 1 = Okavango, 2 = Ngamiland, 3 = Chobe, 4 = Tutume, 5 = Boteti, 6 = Bobirwa.

The use of the KAPs approach was employed to assess what human communities know about mosquito bio-ecology and mosquito-borne infections, attitudes towards the vectors and associated pathogens and their knowledge on control measures. For this study, “Knowledge” refers to what members of the communities know about mosquito biology, taxonomy, ecology and control. “Attitude” refers to individual’s feelings and preconceived ideas (perceptions) about mosquitoes and mosquito-borne infections whilst “Practices” refer to what respondents do to curb mosquito impacts and manage associated disease(s). The interactions between the dependent variables (KAPs) were developed and measured against the independent variables (sub-district, village, sex, age, education and profession) targeting communities in non-endemic (Serowe and Palapye) and endemic (Bobirwa) zones of the Central district, Botswana (Figure 1). Quantification of KAPs was achieved using ordinal and rating scales, following modifications from Machekano et al. [47].

### 2.2. Study Area

Botswana has 10 major districts, six of which are characterised as malaria endemic ([48]; Figure 1A,B). Each district is comprised of various sub-districts, with some districts housing both malaria endemic and non-endemic sub-districts. Three endemic and three non-endemic sub-districts make up the Central district (Figure 1C), offering an opportunity to assess within-district KAP’s in relation to malaria endemicity status. The study was conducted in three areas within the semi-arid Central district of Botswana, namely: Serowe, Palapye and Bobirwa ([49]; Figure 1C). The area is dominated by savanna vegetation with temporary clay lined and rockpool wetlands [50] as potential breeding sites for mosquitoes. Mosquito diversity in the Central district is poorly explored, especially non-anopheline species, due to skewed focus towards malaria vectors. As such, only vectors belonging to *Anopheles gambiae* and *funestus* complexes have been the focus of mosquito studies in the region [29]. Serowe and Palapye sub-districts are deemed non-endemic malaria areas, whilst Bobirwa falls within the malaria endemic zone of the country [41]. Seven villages were identified for the KAP survey in each sub-district according to their geographic distribution. 

### 2.3. Sampling Technique

A structured questionnaire was used to conduct face-to-face interviews with residents of Serowe (*n* = 206), Palapye (*n* = 202) and Bobirwa (*n* = 203) between July and August 2019 using fourth household approach to standardise data collection across study sites (*n* = 611). The questionnaire had four sections namely the socio-demographics, knowledge, attitude and practices on mosquitoes and their infectious diseases (Figure S1). Although live specimens/pictures were not used as interview guides, the questionnaire had direct questions used to assess baseline information from the respondents. Sections had nominal scales, closed and open-ended answer questions on mosquito KAPs. The open questions were post-coded to quantify the responses for analysis. A pre-run trial of questionnaires (*n* = 20) was conducted in Palapye village to revise and refine the questionnaire. Following pre-run and questionnaire “debugging”, interviews were conducted by trained enumerators using both English and Setswana (local language) following e.g., [47,51].

Prior to questionnaire administration, permission was sought from the Ministry of Environment, Natural Resources Conservation and Tourism (Botswana). In addition, the Department of Biological Sciences and Biotechnology, Botswana International University of Science and Technology, approved the survey and provided a covering support letter to seek verbal permission from village leaders (di Kgosi). A written consent was established with participants explaining the survey background, aim, method of data collection and subsequent usage, issues of anonymity and confidentiality. 

### 2.4. Data Analysis

Census and Survey Processing System software (CSPro 7.2) (United States Census Bureau) was used for data entry while, IBM Statistical Package for Social Sciences (SPSS) version 23 was used for statistical analyses. Data were reported in percentages, frequencies and statistically significant variables were separated at 95% confidence interval. Interactions between dependent and independent variables were enunciated using the Pearson Chi-square test of association, and Pearson’s correlation coefficient was used to test for correlation.

## 3. Results

### 3.1. Socio-Demographic Characteristics

The majority of the respondents were literate (88.7%), females (71.5%), able-bodied (91.7%), single (70.4%) and middle aged (30–39 years; (23.6%)) (Table 1). Education level was mostly junior certificate (2 years post-secondary education) (29.5%) or primary school (29%); very few respondents had attended senior secondary (five years post-secondary education) (14.5%), vocational training (8.7%) or tertiary education (4.7%) (Table 1). Information on mosquitoes and mosquito-borne infections was mostly accessed through audio–visual media (e.g., radio/ television) (50.9%), local health facilities (33.9%), family/ friends (4.7%), own experience (4.1%), print media (e.g., newspapers, magazines) (1.8%), electronic sources (0.3%) and other unspecified means (4.3%). 

**Figure 2:**
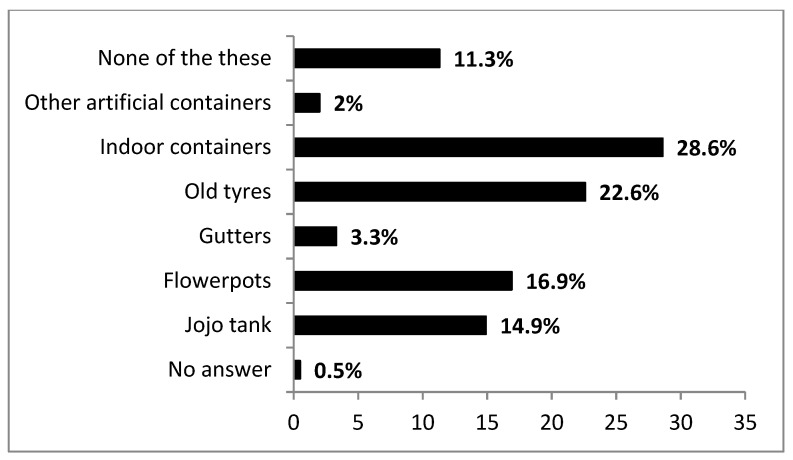
Details of the structure and diversity of water holding containers (outlined by respondents), that may serve as potential mosquito breeding sites found on the premises of respondents across sub-districts.

Fewer households had proper drainage systems (28.9%) compared to those without (71.1%). Conversely, the majority of households had pit latrines (84.5%) exhibiting signs of mosquitoes (e.g., presence of adults, flight or sound) (71.3%) as opposed to those without pit latrines (15.5%) (Table 1). Moreover, households with pit latrines across sub-districts were not significant (χ^2^ = 450, df = 2, *p* = 0.799). Potential vector breeding habitats found in respondents’ property were mainly indoor containers (28.6%), old tyres (22.6%), flowerpots (16.9%), bulk water tanks (Jojo) (14.9%), gutters (3.3%) and other artificial containers (2%) (Figure 2). 

### 3.2. Knowledge

**Figure 3:**
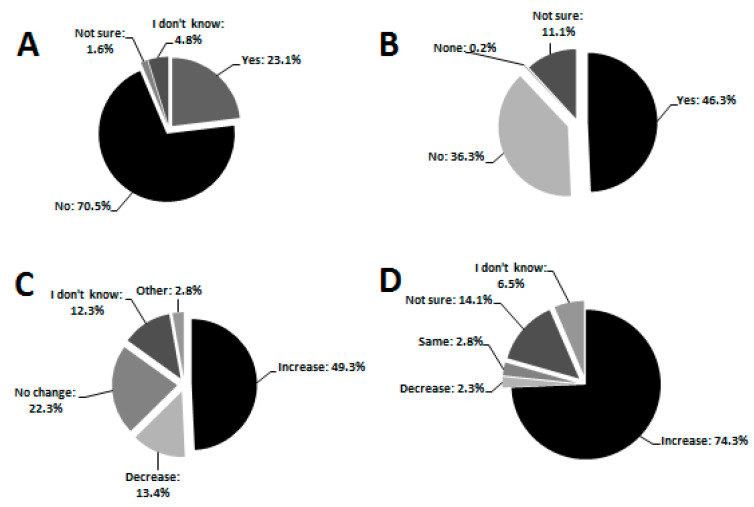
Pie charts summarising community’s knowledge on (**A**) mosquito developmental stages, (**B**) HIV transmission by mosquitoes, (**C**) general mosquito trends over the last 10 years and (**D**) perceived effect of cattle-dung contaminated water on mosquito abundance.

The majority of the respondents knew adult mosquitoes (98.9%) as opposed to those who did not (1.1%). This result was the same across all three sub-districts (χ^2^ = 3.081, df = 2, *p* = 0.214). A total of 99%, 96.5%, 99% had not seen mosquito eggs, larvae and pupae, respectively, and many (70.5%) did not know that mosquitoes go through different developmental stages regardless of the sub-district (χ^2^ = 1.288, df = 6, *p* = 0.972, Figure 3A). During the austral winter (April, May, June 2019), precedent to the current study, the majority of respondents indicated having seen mosquitoes within their properties (54.3%). Similarly, the austral summer prior to our survey (October 2018 to March 2019), the majority (87.4%) indicated having seen and been bitten by mosquitoes too often to be counted (40.0%). Conversely, in austral winter (April to July 2019), the majority of respondents (58.1%) reported no bites while relatively few received very few bites (42.0%) and this differed significantly across the sub-districts (χ^2^ = 63.812, df = 6, *p* < 0.001) with many having received no bites in non-endemic areas. Spatially, the bites were either received both indoors and outdoors (52.2%), indoors (38.8%), outdoors (8.5%) or respondents were not sure (0.5%). Temporally, bites were reported more intensive in the evenings (dusk) (56.3%) followed by night times (36.7%) when in bed. Respondents reported being bitten mostly on the face (39.3%) followed by the arms (34.2%). Furthermore, the majority of respondents believed that mosquito bites could transmit human immunodeficiency virus (HIV) to human beings (46.3%) (Figure 3B) and this was not significantly different across sub-districts (χ^2^ = 9.511, df = 8, *p* = 0.301). The majority of respondents did not know mosquito-borne diseases such as yellow fever (83.3%), dengue (100%), avian malaria (97.5%), and elephantiasis (88.1%), as opposed to those who did not know malaria (2.1%). A total of 72.7% knew the signs and symptoms of malaria but this differed significantly across sub-district (χ^2^ = 42.744, df = 12, *p* < 0.001) with endemic area more knowledgeable than non-endemic. Relatively few respondents had personally suffered malaria (9.3%) but this also differed significantly across sub-districts (χ^2^ = 28.724, df = 6, *p* < 0.001) with non-endemic having less individuals. Although the majority of respondents (67.4%) did not know anyone within their area who had suffered from malaria, a significant proportion (30.8%) did.

Within localities, the majority of respondents (73.6%) confirmed that there were no new types of mosquitoes known to them. The association between the knowledge of mosquitoes and the observation of any knew types of mosquitoes was significant (χ^2^ = 18.827, df = 9, *p* = 0.027). Although the majority did not know if certain parts of their village had more mosquitoes than others (40.9%), a considerable number of respondents (33.6%) reported distribution disparity with certain areas housing more mosquitoes than others. The association between knowledge of mosquitoes in certain parts of the village and the sub-district was, however, significant (χ^2^ = 14.108, df = 6, *p* = 0.028). Thus, localities with institutions such as schools, clinics, camp sites and central business districts (CBD), were believed to attract mosquitoes mainly through availability of stagnant waters (41.0%), dirty environments (8.8%), drainage systems (8.3%), dense vegetation (6.8%) amongst others. Most respondents did not know natural methods of controlling the mosquito population (e.g., use of predators) in adults (82.2%) and juveniles (90.5%). 

The majority of the respondents (49.3%) perceived mosquito abundance to be increasing over the last 10 years (Figure 3C), particularly during summer when temperatures were very high (90.5%). Some responders reported that mosquitoes were present regardless of temperature (6.5%), whilst others were either not sure (1.1%) or did not know (0.5%) about mosquito population trends within their localities. Similarly, the majority identified temperature as the main contributing factor to mosquito proliferation (35.9%).

The majority confirmed cattle to be roaming around their areas (91.5%), particularly free-range cattle reared in the unfenced communal areas (93.8%). As such, most respondents (47.5%) associated the interaction between cattle and the environment as the key contributor to high numbers of mosquitoes. Cattle waste products (e.g., dung and/urine) in water sources were believed to attract mosquitoes by the majority (72.3%). Similarly, 74.3% also believed that cattle-induced eutrophication through dung could modulate the increase in mosquito breeding and abundance (Figure 3D). However, this perception was not significantly associated with specific sub-districts (χ^2^ = 10.428, df = 8, *p* = 0.236). 

### 3.3. Attitude 

**Figure 4:**
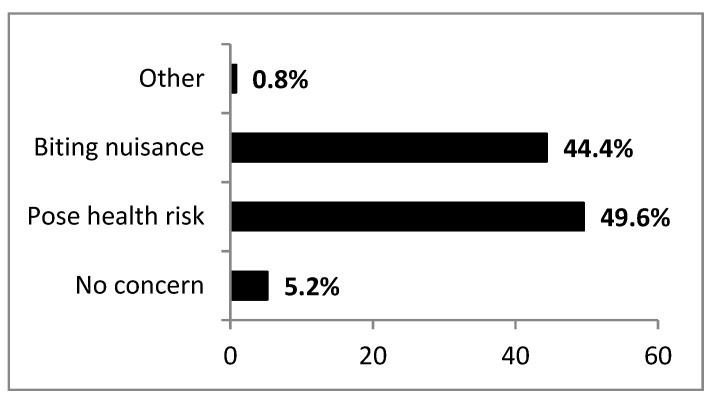
A summary of community concerns on the public health significance of mosquitoes across localities.

A significant proportion of respondents (49.6%) perceived mosquitoes as health risks to the community; 44.4% considered them to be just a biting nuisance while some (5.2%) were not concerned with mosquitoes at all (Figure 4). 

The association between public health significance of mosquitoes and sub-district was, however, significant (χ^2^ = 20.323, df = 6, *p* = 0.002) with the non-endemic area of Palapye mostly (51.8%) concerned about health risks. Whilst most of the respondents affirmed that one mosquito bite could pose a health risk (52.9%), 35.8% did not believe this notion. A small portion of respondents (10.5%) were not sure while even fewer respondents (0.8%) said mosquito bites never pose a health risk with overall no significant differences across sub-districts (χ^2^ = 4.973, df = 6, *p* = 0.547). However, this belief was not linked with the respondent’s level of education (χ^2^ = 20.790, df = 21, *p* = 0.472). In rating concerns about mosquito-borne infections, 23.9% were strongly concerned, 28.5% concerned, 28.2% less concerned, while 19.5% were not concerned. The level of individual concerns over mosquito-borne infections varied significantly across sub-districts (χ^2^ = 52.632, df = 6, *p* < 0.001) with greatest concerns emanating from the non-endemic area of Serowe. Most respondents from the non-endemic area (Palapye) strongly agreed with the notion that cross-border trading traversing sub-districts had the risk of importation of mosquito-borne infections (48.3%) with highly significant association at sub-district level (χ^2^ = 53.625, df = 8, *p* < 0.001). 

### 3.4. Practices

**Figure 5:**
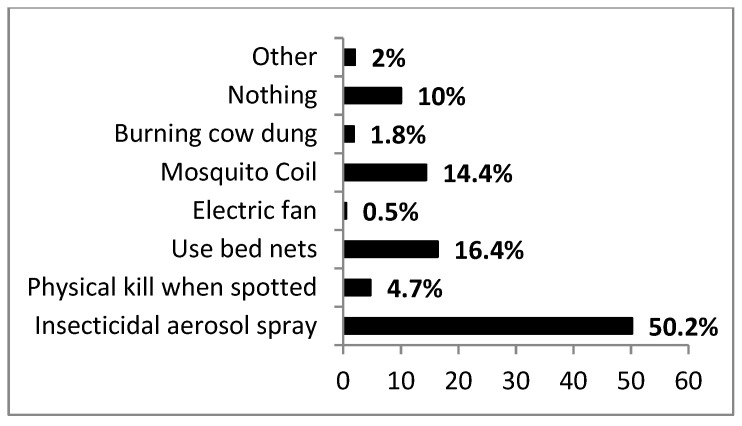
Summary responses (%) on how respondents protect themselves from indoor mosquito bites across sub-districts.

There was some variation on how respondents regarded mosquito bite prevention strategies. Most respondents reported wearing clothes that cover much of the body (38.0%); 22% did nothing; 20% stayed indoors when mosquitoes were active (e.g., from dusk); 10% used repellent products while 9.4% used unspecified means. To discourage mosquito breeding and resting habitats within household properties, the majority of respondents cleared their premises of any grasses/resting plants (73.3%) with the endemic area having the highest response (90.1%) on this practice. Stagnant water in premises was marginally significant across sub-districts (χ^2^ = 12.495, df = 6, *p* = 0.052) and was reported in fewer yards (13.4%) as opposed to those without (85.8%). Moreover, activity of mosquitoes on stagnant water was not significant across sub-districts (χ^2^ = 4.314, df = 6, *p* = 0.634). For indoor intervention, the majority used insecticidal sprays (50.2%) (Figure 5). The endemic area used insecticidal sprays less compared to the non-endemic. Most respondents regarded the use of bed nets as the most effective method of indoor protection (59.9%). Nonetheless, the association between bed net usage and the sub-district was highly significant (χ^2^ = 72.127, df = 12, *p* < 0.001) with the endemic area displaying their highest usage. 

The control of mosquitoes through IRS was noted in communities of the endemic Bobirwa sub-district as a national intervention strategy where the majority did not know the chemical used for IRS in their structures (88.3%). However, the majority (79.6%) testified that IRS was effective in controlling indoor resting mosquitoes. Accordingly, after IRS activity, many (76.9%) observed reductions in the mosquito population indoors and subsequent decreases in biting intensity (71.5%). Lack of knowledge on mosquito predators was not linked to overall access to information on mosquitoes (χ^2^ = 16.219, df = 12, *p* = 0.181). Most respondents (55.3%) regarded the pepper tree (*Schinus molle*) as an effective repellent for adult mosquitoes while many (75.8%) did not know any indigenous/exotic plant capable of treating mosquito-borne diseases. Accordingly, many in the endemic area confirmed sufficient diagnosis and treatment of mosquito-borne infections (59.4%) within their villages by the clinics/hospitals but this varied significantly among sub-districts (χ^2^ = 50.991, df = 6, *p* < 0.001). A total of 65.1% indicated that they had travelled to malaria endemic districts mostly for more than two weeks (74.1%). The majority of respondents (90.2%) did not take any prescribed prophylactic medication against mosquito-borne infections, either as residents in, or when travelling to malaria-endemic areas.

## 4. Discussion

Our study showed that the majority of household respondents were females. According to the Afro-cultural norm, men go out to work to generate income for family support while women stay home and engage in day-to-day household activities [52,53]. Women are therefore regarded vulnerable to vector-borne diseases although they are strong drivers of rural household welfare [54]. The most common source of information on mosquitoes and mosquito-borne infections was through audio–visual media (e.g., radio/television) with non-endemic areas having greater information access than endemic areas. Information access remains a challenge to the remote and unserviced (e.g., no electricity, mobile/telephone network range and television reception) endemic areas, particularly with individuals who cannot afford to acquire audio–visual media especially the poor and socially vulnerable [55]. This is highly critical, although the knowledge gap on mosquito-borne diseases was not linked to how information was accessed (χ^2^ = 15.841, df = 18, *p* = 0.604). Endemic areas may constitute groups highly vulnerable to vector-borne diseases, necessitating the need for intensive education and communication across urban–rural gradients [56]. 

Surveyed premises revealed uniform usage of pit latrines across sub-districts. Furthermore, the majority of respondents confirmed that pit latrines facilitated mosquito presence. Although pit latrines are the most cost-effective ablution facilities in low income rural settlements, they significantly contribute to direct and indirect compromised household hygiene. For example, they provide mosquito refugia, oviposition sites and food resources for juveniles (e.g., larvae) [57]. Most nuisance homestead mosquitoes (e.g., *Anopheles* and *Culex* sp.), utilise these pit latrine as “hotspots” for successful propagation [58] and further leverage other human-health complications (see discussions in Nakagiri et al. [57]. Given this scenario, modified mosquito proofing structures that limit mosquito entry into the pit latrines and minimise potential breeding may be better explored [59,60,61]. Similarly, drainage systems contribute significantly as a habitat for mosquito species providing diverse nutrient inputs to developing juveniles [62]. Although few respondents had proper drainage systems within their properties, caution is needed on their regular maintenance (e.g., overflow avoidance, entry point elimination) across sub-districts. In addition, old tyres had a considerable proportion (22.6%) of potential water holding within premises mostly in endemic areas. These microhabitats are highly favourable to aedine species [63] given that their colonisation and abundance in differential landscapes mainly revolve around human-mediated environmental modifications [64]. 

Although the majority of respondents reported knowledge of adult mosquitoes, lack of recognition on mosquito juveniles was evident with similar trend across endemic and non-endemic areas irrespective of location (sub-district) (*p* = 0.972), education level (*p* = 0.094) or literacy (*p* = 0.681). The knowledge of these bionomic processes may deepen the community’s understanding and enhance life-stage specific intervention strategies especially on the larvae that colonise many container-type microhabitats [65] that are common at most visited homesteads. Most respondents who confirmed knowledge of adult mosquitoes, identified them through their “sound” as opposed to gross morphology and simplified taxonomic identification keys (0.2%). Regardless of the endemicity status, the local communities need these identification keys and skills to identify mosquito species capable of transmitting debilitating diseases in humans, wildlife and livestock [6,12,66,67]. The current study relied on respondents’ assumptions that indeed they know and/or have seen mosquitoes within their homesteads. Therefore, to err on the side of caution, the conclusions drawn from this study should be interpreted within the framework of this limitation. Moreover, the current study did not perform independent assessment of mosquito habitats and mosquitoes at each residence. Similar future work should thus make independent assessments of information (e.g., presence and absence of mosquito larval habitats, mosquitoes and developmental stages) to complement questionnaire data. In addition, no mosquito abundance data were available for the study sites and so we could not assess if KAPs were in any way related to fine-scale exposure to mosquito burden. We thus recommend improved approaches of administering questionnaires using pictorial aids to effectively assess knowledge.

With the exception of malaria, all sub-districts exhibited very limited knowledge on other mosquito-borne infections. The result is in keeping with previous studies to date [12,68]. This could be due to the bias of the burden of malaria under prevailing climatological impacts locally [69] and its over-representation at global scale [70,71]. Our results also showed mosquito summer bites increment with a similar trend across sub-districts, although highly variable in winter. Again, the endemic area experienced the greatest bites in winter compared to the non-endemic. Thus, studies reported active mosquito-borne transmission shifts that are climate modulated [72] necessitating spatio-temporal supplementary vector control interventions. Further research is, therefore, needed on seasonal mosquito population and biting dynamics at national level. Moreover, modelling disease and vector status remains crucial given the increased mosquito population trends over the last 10 years (49.3%) as mostly reported in non-endemic areas (Palapye). Global change may increase mosquito numbers and parasite virulence through climate-mediated influences [33]. Temperature plays a crucial role in facilitating shortened mosquito life cycle and increased generations/year [73]. Furthermore, consistent with respondents’ observations, there is need to investigate the contribution of cattle-induced eutrophication on the aquatic ecosystem regarding impacts on mosquito proliferation and implication on natural predators. Aquatic predators may potentially play a critical role in community structure assemblage as a sustainable biocontrol tool if utilised effectively in the local context [74,75].

The majority of the respondents confirmed mosquitoes pose health risks within their communities, although different sub-districts varied significantly on this assertion. The non-endemic area of Palapye had the highest concerns of health risks necessitating special attention. The study areas were all affected differentially by mosquito-borne infections, e.g., malaria (*p* = 0.019). The burden of malaria gained ground especially in the endemic area (Bobirwa) more than any other mosquito-borne infection. Recently, areas outside the endemic areas previously not known to have indigenous cases of malaria, reported more sporadic cases including the imported cases across the country [69]. Moreover, through travel and networking, imported cases may be guaranteed given the prevalence of other life-threatening mosquito-borne infections in the neighbouring countries regionally [76] and elsewhere [77,78]. Accordingly, non-endemic study sites are at risk since they are an intersection of many risk factors [79,80]. This calls for community mobilisation in both endemic and non-endemic zones in raising awareness not only for malaria but all other mosquito-borne related illnesses [81]. While it is encouraging that most communities asserted that one mosquito bite could lead to health risk, there is more to be done in ensuring further development and bridging knowledge gaps in parasite transmission blockage dynamics [82], more so in light of the respondents’ request to be trained on mosquito biology (38.5%). In addition, the results of the survey showed that mosquito bites were mostly received both indoors and outdoors (52.2%) possibly relating to human behaviour [83] and the resting patterns indoors (endophilic) and outdoors (exophilic) influenced by microclimatic factors [84]. Thus, more work is also needed in investigating mosquito biting-patterns, useful in determining appropriate interventions against mosquito vectors [85]. Further, community willingness to effectively explore both indoor and outdoor interventions is highly desirable in arresting mosquito life-stage development and survival mechanisms. 

Although the majority of respondents use clothes that cover much of the body for personal protection [86], this practice differed significantly across sub-districts (*p* < 0.001), with endemic areas exhibiting the highest personal protection compared to the non-endemic areas. A higher proportion reported face bites (39.3%) suggesting that there is a need to explore other protection measures that may cater for exposed areas (e.g., head). Thus, further knowledge and awareness regarding other methods of personal protection other than long sleeved clothing (e.g., acoustic and electric devices) are essential [87,88]. The bed nets were regarded as an overall effective indoor intervention against adult mosquitoes, although mostly used in the endemic area. Conversely, insecticidal aerosol spray use dominated more than bed nets in the non-endemic areas. The use of aerosol sprays together with the national intervention strategies (e.g., IRS and LLITNs in endemic areas) may exacerbate resistance in mosquito species both in the endemic and the non-endemic areas. Whilst the ongoing “more than one” chemical-based intervention [89] and prolonged pesticide use on mosquitoes are practiced, susceptibility status assessing potential resistance development remains eminent. The communities need to understand issues of induced resistance at household level through organised campaigns and public education as a way of managing resistance in disease vectors. This will augment onto the overall holistic approach of appropriate practices involving personal, indoor and environmental manipulations (e.g., clearing resting and foraging vegetation) as demonstrated by most respondents (73.3%) in the endemic area [90].

The respondents reported cattle within their areas across all sub-districts highlighting that more work may be needed to determine the role of animal-induced nutrification in wetland systems as a factor driving mosquito abundance. Degraded aquatic habitats are known to negatively impact useful natural enemies of mosquito populations [91]. As such, further research on degraded aquatic ecosystems may be useful in advising communities on cattle movement, herd size and managing watering points. This may reduce mosquito proliferation in villages that have natural ponds and numerous other water-collecting structures while conserving natural enemies as a tool for ecosystem service [40,92]. Exotic and indigenous plant species outlined as attractants and/ or repellents for mosquitoes need further exploration. The outcomes may optimise traditional knowledge systems (locally available plants) in sustainable vector control measures and advise the communities on their utilisation (e.g., push–pull vector control systems) [93]. The majority of the respondents reported having travelled to the endemic areas without prophylactic treatment [94], mostly those in the endemic area (Bobirwa). Communities should be constantly advised to take medication regardless of endemicity status as drugs strengthen immunity and further block mechanisms of parasite replication [95].

## 5. Conclusions

Results of this survey indicate that the majority of the community had limited knowledge in many areas of mosquito bio-ecology. Although most were concerned about contracting mosquito-borne infections, capacitating the communities on awareness of personal, structural and environmental control strategies through public education is needed. Furthermore, it is essential to educate communities on practices that control mosquito populations without harming the environment. These include management of water holding structures (e.g., pit latrines, drainage), chemical intervention and promoting and conservation of natural enemies needed for sustainable integrated control of mosquito populations, with consequent reduction of associated disease burdens. The knowledge paucity reported here points to a need for training of local communities in mosquito bio-ecology, especially identification of key developmental life stages for efficient vector and disease management enhanced by modern real-time “citizen science” application as a reporting system for prompt vector surveillance initiatives. This may help in early warning systems against the spread of vectors and associated pathogens and the management of emerging and re-emerging mosquito-borne infections under anthropogenic changing environments. 

## Figures and Tables

**Table 1:** Summary results of the socio-demographic characteristics for non-endemic areas (Serowe and Palapye) and endemic (Bobirwa) sub-districts (*n* = 611).

Variables	Category	Number of Respondents	Proportion (%)
Gender	Male	174	28.5
	Female	437	71.5
Marital Status	Single (never married)	430	70.4
	Married	122	20
	Divorced	9	1.5
	Widowed	50	8.2
Age (years)	18–29	135	22.1
	30–39	144	23.6
	40–49	110	18.0
	50–59	89	14.6
	≥60	133	21.8
Disability	Yes	49	8.0
	No	560	91.7
	Prefer not to say	2	0.3
Literacy	Literate	542	88.7
	Illiterate	65	10.6
	Prefer not to say	4	0.7
Education	None	83	13.6
	Primary	177	29.0
	Junior Certificate	180	29.5
	Form 4–5 (Senior)	90	14.7
	Vocational	50	8.2
	Tertiary	29	4.7
	Prefer not to say	1	0.2
	Other	1	0.2
Information access	Radio/TV	311	50.9
	Health professionals	207	33.9
	Printed media	11	1.8
	Electronic sources	2	0.3
	Family/Friends	29	4.7
	Own experience	25	4.1
	Other	26	4.3
Family size	1–2	84	13.7
	3–5	197	32.2
	6–10	229	37.5
	>10	101	16.5
Pit latrine (toilet)	Yes	516	84.5
	No	95	15.5
Drainage system	Yes	176	28.9
	No	433	71.1
Stagnant water	Yes	82	13.4
	No	524	85.8
	Not sure	4	0.7
	Don’t know	1	0.2

## Supplementary Materials

The following are available online at https://www.mdpi.com/1660-4601/17/21/8196/s1, Figure S1: Mosquito Knowledge, Attitude and Practices by Communities in the Serowe, Palapye and Bobirwa Sub-districts, Botswana. Household Questionnaire.
