# Supplementary material for: Are Vulnerable Communities Thoroughly Informed on Mosquito Bio-Ecology and Burden?
Source: Int J Environ Res Public Health. 2020 Nov 6;17(21):8196. doi: 10.3390/ijerph17218196 (PMC7672552; doi:10.3390/ijerph17218196)
Supplement: Supplementary file 1 [file ijerph-17-08196-s001.pdf]

Questionnaire Code

Questionnaire number	*Interviewer Initials*	*Sub-district*

BIUST
BOTSWANA INTERNATIONAL UNIVERSITY OF SCIENCE & TECHNOLOGY
2019

Mosquito Knowledge, Attitude and Practices by Communities in the Serowe, Palapye and Bobirwa Sub-districts, Botswana.

Household Questionnaire

GENERAL

**Respondent name(s):**	**Surname:**____________________________________ **First name:** __________________________________
**Date :**	_______/________/ ________
**Interviewer Name:**	___________________________ **Interviewer ID**____________________
**Location ID :**	**Sub-district:** ___________________________________ **Village/ Town:** ___________________________________ **GPS Coordinates** ___________________________________

SECTION 1.0 DEMOGRAPHICS

**1.1**	What is your gender?<br>1= Male, 2= Female	
**1.2**	What is your age range?<br>1=18- 29 years, 2=30-39 years, 3=40- 49 years, 4= 50- 59 years, 5= ≥60 years	
**1.3**	What is your marital status?<br>1= Married, 2= Divorced, 3= Widowed, 4= single (never married),<br>5= Other (specify)…………………………………………	
**1.4**	Are you disabled?<br>1= Yes, 2= No, 3= Prefer not to say	
**1.5**	Are you literate?<br>1= Yes, 2= No, 3= Prefer not to say	
**1.6**	What is your highest level of education?<br>1= None, 2= Primary, 3= JC, 4= Form 4-5 (senior), 4= vocational, 5= Tertiary, 6= Prefer not to say, 7= Other (Specify)……………………………………………	

**1.7**	What is your profession?<br>1= Farmer, 2= Self-employed, 3= Entrepreneur, 4= Employee, 5= Not working, 6= Other (Specify)………………………………………….	
**1.8**	How do you access information about mosquitoes and mosquito-borne diseases? (May select more than one).<br>1= Audio/visual source (Radio/TV), 2=Health professionals, 3=Print media (newspapers and others), 4= Electronic sources (Cellphone, computers and others) 5= Friends/ family/ relatives, 6 = Own experience, 7 = Other (Specify)…………………………………………………..	
**1.9**	How many of you are staying on the property?<br>1= 1-2, 2= 3-5, 3= 5-10, 4= >10	
**1.10**	Which of the following are found in your property? (May give more than one)<br>1= JoJo tank, 2= flowerpots, 3 = gutter, 4 = old tyres, 5= indoor containers, 6= None of the above, 7= Other artificial containers (Specify)……………………………………………….	
**1.11**	If your answer is 2 in 1.10, where are the flowerpots located? (If no, continue to 1.12)<br>1= In the house, 2= Outside in the veranda/ porch/ terrace/ balcony, 3= Outside around the house, 4= Outside distant from the house, Other (Specify)…………………………………….	
**1.12**	Do you have a pit latrine in your yard? (If no, continue to 1.15)<br>1= Yes, 2= No	
**1.13**	If yes to 1.12, are there signs of mosquitoes in it?<br>1=Yes, 2=No, 3= Not sure, 4= Don't know	
**1.14**	Do you think it encourages mosquito breeding?<br>1=Yes, 2=No, 3= Not sure, 4= Don't know	
**1.15**	Is there a drainage system in your yard? (If no, continue to 1.18)<br>1= Yes, 2= No	
**1.16**	If yes to 1.15, do you think it has an effect on mosquito breeding?<br>1=Yes, 2=No, 3= Not sure, 4= Don't know	
**1.17**	Are there signs of mosquitoes in the drainage system?<br>1=Yes, 2=No, 3= Not sure, 4= Don't know, 5= Did not check	
**1.18**	Do you have stagnant waters in the yard, either 'natural'/artificial? (If your answer is 2,3 or 4, continue to 2.1)<br>1=Yes, 2=No, 3= Not sure, 4= Don't know	
**1.19**	If yes to 1.18, are there signs of mosquito activity?<br>1=Yes, 2=No, 3= Not sure, 4= Don't know	

SECTION 2.0 KNOWLEDGE

**2.1**	Do you know mosquitoes? 1= Yes, 2= No, 3= Not sure, 4= I don't know	

**2.2**	How do you identify them?<br>1= Colour, 2= Size, 3= Sound, 4= body parts, 5= Taxonomy key, 6= Specify……………………	
**2.3**	How many types of mosquitoes do you know? 1=0, 2=1, 3=2, 4=3, 5=4, 6= >5	
2.4	Can you identify the different types of adult mosquitoes?<br>1= Yes, 2= No, 3 =Not sure, 4= Don't know	
**2.5**	Have you seen an adult mosquito in the last 3 months?<br>1= Yes, 2= No, 3= Not sure	
**2.6**	If yes to 2.5, what is the colour of the mosquito(es) you have seen?<br>1= Light brownish, 2= black and white, 3= Not sure, 4= Other (Specify)…………………………	
**2.7**	Do you know mosquitoes go through different developmental stages?<br>1= Yes, 2= No, 3= Not sure, 4= I don't know	
**2.8**	Have you ever seen the following developmental stages of mosquitoes apart from adults? (If no to all, continue to 2.10)<br>1. Eggs Yes………………………No…………………………<br>2. Larvae Yes……………………..No…………………………<br>3. Pupae Yes……………………No…………………………..	
**2.9**	If yes to 2.8, where did you see them?<br>………………………………………………………………………………	
**2.10**	Did you experience a mosquito bite in the last summer (October to March) in your village?<br>1= Yes, 2= No, 3= None, 4= Not sure, 5= Don't know	
**2.11**	If yes to 2.10, how many bites in a day?<br>1= Too many to count, 2= Many, 3= Moderate, 4= Few, 5= Very few	
**2.12**	Did you experience in your village, a mosquito bite in winter (April to July)?<br>1= Yes, 2= No, 3= None, 4= Not sure, 5= Don't know	
**2.13**	If yes to 2.12, how many bites in a day?<br>1= Too many to count, 2= Many, 3= Moderate, 4= Few, 5= Very few	
**2.14**	Where did you get bitten by the mosquito? 1= Indoors, 2= Outdoors, 3= Both indoors and outdoors, 4= I don't know	
**2.15**	What time of the day did you normally experience mosquito bites?<br>1= Morning, 2= Noon, 3= Afternoon, 4= Evening, 5= Night, 6= Other (Specify)………………	
**2.16**	Which part of the body received lots of bites?<br>1= head, 2= neck, 3= chest, 4= back area, 5= arms, 6= legs, 7= Other (Specify) ………………	
**2.17**	Do you think mosquitoes can transmit HIV during blood feed?<br>1= Yes, 2= No, 3= None, 4= Not sure, 5= Don't know	

**2.18**	What time of the year are mosquitoes abundant? (May choose more than one)<br>1= Winter, 2= Spring, 3= Summer, 4= Autumn, 5= All year round, 6= Not sure, 7= Other (Specify)…………………………	
**2.19**	Do mosquitoes get plenty when it is hot or cold?<br>1= Hot, 2= Cold, 3= Both Hot and Cold, 4= Not sure, 5= Don't know	
**2.20**	In your opinion, what is the general trend of mosquito abundance over the past 10yrs?<br>1= increasing, 2= decreasing, 3= No change, 4= Don't know	
**2.21**	If your answer to 2.20 is 1, what do you think is the main contributing factor?<br>1= Temperature, 2= Precipitation, 3= hosts, 4= I don't know, 5= Other (Specify)……	
**2.22**	Which diseases do you think mosquitoes may spread?<br>………	
**2.23**	Do you know signs and symptoms of any diseases you mentioned?<br>1= Yes, 2= No, 3= None, 4= Not sure, 5= Don't know	
**2.24**	If yes in 2.23, state one disease and its signs and symptoms.<br>Disease……	Signs & symptoms
**2.25**	Do you know:<br>a (i) Yellow fever? 1=Yes, 2=No, 3= Not sure, 4= Don't know……………………………………….<br>a (ii) what vectors it ……………………………….and its parasite?……………………………………..<br>b (i) Dengue fever? 1=Yes, 2= No, 3= Not sure, 4= Don't know…………………………………….<br>b (ii) what vectors it ……………………………….and its parasite?...<br>c (i) human malaria? 1=Yes, 2= No, 3= Not sure, 4= Don't know…………………………………..<br>c (ii) what vectors it ……………………………….and its parasite?...<br>c (iii) how is it diagnosed locally? ………………………………………………………………………………<br>d (i) Elephantiasis? 1=Yes, 2=No, 3= Not sure, 4= Don't know……………………………………..<br>d (ii) what vectors it ……………………………...and its parasite?……………………………………..<br>e (i) avian malaria? 1=Yes, 2= No, 3= Not sure, 4= Don't know…………………………………….<br>e (ii) what vectors it? ……………………………..and its parasite?……………………………………..	
**2.26**	Have you ever suffered from a mosquito-borne disease?<br>1=Yes, 2=No, 3= Not sure, 4= Don't know	
**2.27**	Do you know anyone who suffered from a mosquito-borne disease?<br>1=Yes, 2=No, 3= Not sure, 4= Don't know	
**2.28**	If your answer to 2.26 and 2.27 is yes. Which diseases?	

	……	
**2.29**	Are there any new types of mosquito species in your area that were not here before?<br>1=Yes, 2=No, 3= Not sure, 4= Don't know	
**2.30**	Do you have cattle in your area/ village?<br>1=Yes, 2=No, 3= Not sure, 4= Don't know	
**2.31**	If yes to 2.30, are there reared in fenced farms or unfenced communal areas?<br>1= Fenced farms, 2=Unfenced communal areas, 3= Not sure, 4= Don't know	
**2.32**	As far as Cattle interact with the environment, do you think this may have an effect in mosquito numbers?<br>1=Yes, 2=No, 3= Not sure, 4= Don't know	
**2.33**	Do you think cattle dung (and other waste products) dropped in water bodies can be a good resource for breeding mosquitoes?<br>1=Yes, 2=No, 3= Not sure, 4= Don't know	
**2.34**	If water is contaminated with dung, what effect do you think it has on the numbers of breeding mosquitoes?<br>1= Increase, 2= Decrease, 3= Same, 4= Not sure, 5= I don't know	

SECTION 3.0 ATTITUDE

**3.1**	What do you think about the 'economic' significance of mosquitoes in your area?<br>1= No concern, 2= Pose a health risk, 3= Nuisance 4= Other (Specify) …………………………	
**3.2**	Could one mosquito bite be of any health risk?<br>1=Yes, 2=No, 3=Not sure, 4= Never	
**3.3**	After being bitten by a mosquito, what do you do?<br>1= Nothing, 2= consider protective measures, 3= wait for fever symptoms, 4= visit a clinic, 5= Other (Specify)…………………………………	
**3.4**	How can you rate your concern on a mosquito-borne infection?<br>1= Strongly concerned, 2= concerned, 3= Less concerned, 4= Not concerned	
**3.5**	What do you do to discourage mosquito breeding and resting habitats in your property? (May choose more than one)<br>1= Land clearing, 2=eliminating water holding containers, 3= Nothing, 4= Other (Specify)……………………………………………………………	
**3.6**	What mosquito training do you need?<br>1= Biology, 2= Identification, 3= Ecology, 4= I do not want, 5= Other (Specify)………………	

SECTION 4.0 PRACTICES

**4.1**	Which method do you use to protect against mosquitoes indoors?<br>1= Spray with insecticides, 2= Physical kill when spotted, 3= Use bed nets, 4= Electric fan, 5= Mosquito coil, 6= Burning cow dung, 7= Window gauze 8= Nothing, 9= Other (Specify)…………………………………	
**4.2**	If your answer to 4.1 is either 1, 2, 3, 4, 5, 6 or 7, which one(s) are most effective?<br>………	
**4.3**	How do you and your family protect yourselves from mosquito bites?<br>1= Stay indoors in the evening, 2= Wear long cloths/ sleeves, 3= Apply repellents, 4= Nothing, 5= Other (Specify)................................	
**4.4**	If your answer to 4.3 is either 1, 2 or 3, which one (s) are most effective?<br>……………………………………………………………………………………………………	
**4.5**	Is the government spraying your houses to control mosquitoes? (If no, continue to 4.10)<br>1=Yes, 2= No, 3=Not sure, 4= I don't know	
**4.6**	If yes to 4.5, which chemicals are used for spraying?<br>____	
**4.7**	Do you think indoor residual spraying (IRS) is effective in controlling indoor resting mosquitoes?<br>1= Yes, 2=No, 3= Not sure, 4= I don't know	
**4.8**	After spraying, what happens to mosquito population densities indoors?<br>1= Increase, 2= Reduce, 3= Same, 4= Not sure, 5= I don't know.	
**4.9**	After spraying, what happens to mosquito biting rates?<br>1= Increase, 2= Reduce, 3= Same, 4= Not sure, 5= I don't know.	
**4.10**	Are there certain parts of the village where mosquitoes are more than others?<br>1=Yes, 2=No, 3=Not sure, 4= I don't know	
**4.11**	If yes to 4.10, where and why?<br>………<br>………	
**4.12**	Do you know of any animals/ organisms that eat adult mosquito?<br>1 = Yes, 2=No, 3 = Not sure	
**4.13**	If yes to 4.12, do you know which ones?..	
**4.14**	Do you know any animals/ organisms that eat mosquito juveniles (egg-pupa)?<br>1= Yes, 2=No, 3 = Not sure	
**4.15**	If yes to 4.14, Which ones? ………………………………………………………………………………	

**4.16**	Cross boarder trading that traverse Serowe, Palapye and/or Bobirwa sub-districts have the risk of imported mosquito-borne infections. Do you agree with this notion?<br>1= Strongly agree, 2= Agree, 3= Disagree, 4= Strongly disagree, 5= Don't know	
**4.17**	Do you think cross-border screening of infectious diseases and foreign biological samples effectively carried out?<br>1=Yes, 2=No, 3= Sometimes, 4= Not sure, 5= Never, 6 Don't know	
**4.18**	Have you ever travelled to malaria endemic district/ sub-districts (Okavango, Ngamiland, Boteti, Chobe, Tutume, and Bobirwa? If no continue to 4.21.<br>1=Yes, 2=No, 3= I can't remember	
**4.19**	If yes to 4.18, how long was your stay?<br>1= less than one week, 2= up to two weeks, 3= more than two weeks	
**4.20**	Did you take any prescribed medication to these malaria endemic districts?<br>1=Yes, 2=No, 3= I can't remember	
**4.21**	Do you know any indigenous/ exotic plant that is used to repel mosquitoes?<br>1=Yes, 2=No, 3= Not sure, 4= Don't know	
**4.22**	If yes to 4.21, which one(s)?..	
**4.23**	Do you know any indigenous/ exotic plant that treats mosquitoes-borne diseases?<br>1=Yes, 2=No, 3= Not sure, 4= Don't know	
**4.24**	If yes to 4.23, specify which..	
**4.25**	Is there sufficient and available diagnosis and treatment for mosquito-borne diseases in your locality?<br>1=Yes, 2=No, 3= Not sure, 4= Don't know	
**4.26**	Is information sharing and awareness practices on mosquitoes and control measures sufficient in your area?<br>1=Yes, 2=No, 3= Not sure, 4= Don't know	
**4.27**	If no to 4.26, what can be done to better the situation?<br>1= Public education, 2= Community training, 3= Workshops, 4= Campaigns, 5= Not sure, 6= Other (Specify)...	
